# Comparative analysis of safety and outcomes of Non-intubated versus intubated uniportal video-assisted thoracic surgery using propensity score matching: a single-center experience expanding indications beyond traditional restrictions

**DOI:** 10.3389/fsurg.2026.1798894

**Published:** 2026-04-15

**Authors:** Fahim Kanani, Ingrid Grebneva, Diego González Rivas, Khaled Aotman, Anas Salhab, Rijini Nugzar, Mordechai Shimonov, Firas Abu Akar

**Affiliations:** 1 Department of Surgery, The Edith Wolfson Medical Center, Tel Aviv University, Holon, Israel; 2 Gray Faculty of Medicine, Tel Aviv University, Tel Aviv, Israel; 3 Department of Anaesthesia, the Edith Wolfson Medical Center, Tel Aviv University, Holon, Israel; 4 Department of Thoracic Surgery, Quirónsalud A Coruña Hospital, A Coruña, Spain; 5 Department of General Surgery, Faculty of Medicine, Al-Quds University, East Jerusalem, Palestine; 6 Department of Thoracic Surgery, the Edith Wolfson Medical Center, Tel Aviv University, Holon, Israel

**Keywords:** awake uniportal VATS spontaneous ventilation, non-intubated thoracic surgery, pleural effusion, postoperative complications, propensity score matching, uniportal video-assisted thoracoscopic surgery

## Abstract

**Background:**

Non-intubated uniportal video-assisted thoracoscopic surgery (NI-UVATS) has emerged as an alternative to conventional intubated approaches, yet its applicability across diverse patient populations and procedure types remains undefined. We evaluated perioperative outcomes of NI-UVATS vs. intubated UVATS (I-UVATS) in an unrestricted cohort.

**Methods:**

This retrospective cohort study analyzed 289 consecutive VATS procedures (January 2017–June 2025) at a single center. Patients underwent either I-UVATS (*n* = 166) or NI-UVATS (*n* = 123) based on surgeon and anesthesiologist preference. Primary outcome was serious complications (composite of mortality, reintubation, pneumonia, or reoperation). Secondary outcomes included 30-day mortality, length of stay, and procedure-specific complications. Propensity score matching (1:1) was performed to address baseline imbalances. *Post-hoc* stratification by procedural complexity was conducted.

**Results:**

After propensity score matching, 98 patients in each group were analyzed. Despite matching, significant procedural heterogeneity persisted: anatomical resections comprised 36.7% of I-UVATS vs. 5.1% of NI-UVATS procedures (*p* < 0.001). For low-complexity procedures (*n* = 118), serious complications occurred in 10.8% I-UVATS vs. 7.4% NI-UVATS (*p* = 0.545). For medium-complexity procedures (decortications, *n* = 37), serious complications were comparable (16.0% I-UVATS vs. 16.7% NI-UVATS, *p* = 0.959). The limited number of NI-UVATS anatomical resections (*n* = 5) precluded meaningful comparison for high-complexity procedures. Operative time was longer in NI-UVATS (median 52 vs. 37 min, *p* = 0.042). Overall serious complications occurred in 14.3% I-UVATS vs. 11.2% NI-UVATS patients (*p* = 0.522). Thirty-day mortality was 12 (12.2%) in I-UVATS vs. 7 (7.1%) in NI-UVATS (*p* = 0.240), and surgery-related mortality at 1 year was 10 (10.2%) vs. 15 (15.3%), respectively (*p* = 0.291)

**Conclusions:**

NI-UVATS demonstrated safety and feasibility for low-to-medium complexity thoracic procedures within current real-world selection patterns. The marked procedural imbalance (36.7% vs. 5.1% anatomical resections) reflects contemporary practice where surgeons reserve NI-UVATS for lower-complexity interventions. These findings support NI-UVATS implementation for appropriately selected patients undergoing diagnostic and pleural procedures, while anatomical resections remain predominantly performed under intubation. Procedure-specific randomized trials are needed to define the role of NI-UVATS in complex resections.

## Introduction

Non-intubated uniportal video-assisted thoracoscopic surgery (NI-UVATS) emerged as an alternative anesthetic approach initially reserved for high-risk patients deemed unsuitable for conventional intubated general anesthesia (1–3). Initially designed for patients with severely compromised cardiopulmonary function, NI-UVATS aimed to reduce hemodynamic instability and ventilator-associated complications of mechanical ventilation (3, 4). The rationale centered on preserving spontaneous ventilation through regional anesthesia combined with sedation, thereby maintaining diaphragmatic function and reducing ventilation-perfusion mismatch (5, 6).

As experience accumulated, evidence supporting NI-UVATS expanded beyond its initial high-risk population. Multiple randomized controlled trials and meta-analyses have now evaluated its efficacy across diverse thoracic procedures and patient populations (7–9). A 2021 meta-analysis of 14 RCTs encompassing 1,426 patients demonstrated shorter hospital stays (mean difference: −1.41 days) and reduced postoperative complications with NI-UVATS compared to intubated approaches (8). Similarly, a 2019 meta-analysis of 27 studies including 2,537 patients reported fewer overall complications (OR: 0.505, *P* < 0.001) and accelerated recovery metrics in the NI-UVATS cohort (10). Recent RCTs have further validated these findings, including a 2024 non-inferiority trial confirming comparable postoperative pulmonary complications between techniques (11).

The evolution from minor procedures to major anatomical resections reflects growing confidence in NI-UVATS safety profile. Initial applications focused on pleural biopsies, wedge resections, and sympathectomies, but contemporary series increasingly report lobectomies, segmentectomies, and even pneumonectomies or carina performed without intubation (12, 13). A multicenter propensity-matched analysis of 208 patients undergoing VATS bullectomy demonstrated equivalent morbidity and recurrence rates with shorter operative times and hospital stays favoring NI-UVATS (14).

However, significant limitations persist. The inability to achieve complete lung isolation complicates hilar dissection and lymph node sampling, with studies reporting fewer lymph nodes harvested in NI-UVATS lobectomies (15, 16). Conversion to intubation occurs in 0%–10% of cases, primarily due to hypoxemia, bleeding, or inadequate surgical exposure (17). The absence of positive pressure ventilation limits the ability to reopen collapsed alveoli through recruitment maneuvers, while cough reflexes and mediastinal oscillation challenge surgical precision. Additionally, NI-UVATS demands experienced anesthetic teams capable of rapid airway and hemodynamic management when complications arise (18).

Given these evolving indications and persistent controversies, we conducted this retrospective analysis to evaluate the intraoperative feasibility and perioperative outcomes of NI-UVATS vs. conventional intubated U-VATS across diverse thoracic procedures and clinical scenarios. Our objective was to assess real-world applicability beyond controlled trial settings, examining both technical success and complication profiles in an all-comers population including high-risk patients and malignant pathology.

## Methods

### Study design and population

This retrospective cohort study analyzed all consecutive uniportal video-assisted thoracoscopic surgery (U-VATS) procedures performed at Wolfson Medical Center between January 2017 and June 2025. The study protocol received institutional review board approval (IRB #0083-25-WOMC) with a waiver of informed consent due to its retrospective nature. Reporting followed the Strengthening the Reporting of Observational Studies in Epidemiology (STROBE) guidelines (19). The study was designed as an unrestricted cohort analysis, defined as the inclusion of all consecutive adult patients undergoing elective uniportal VATS regardless of procedure type, disease etiology, or comorbidity burden—including patients traditionally considered high-risk (ASA III–IV) and those with malignant pathology—rather than restricting enrollment to a predefined low-risk or single-procedure population.

All adult patients (≥18 years) were stratified into two cohorts based on anesthetic technique: NI-UVATS and conventional I-UVATS. Inclusion criteria were adapted from established protocols (4, 6, 7) and comprised: (1) elective U-VATS procedures including pulmonary resections (wedge, segmentectomy, lobectomy), pleural procedures, and mediastinal procedures; (2) American Society of Anesthesiologists (ASA) physical status classification I-IV; (3) complete perioperative records including anesthetic management details and 90-day follow-up data.

Exclusion criteria aligned with published contraindications for NI-UVATS (3, 5) and included: (1) emergency procedures; (2) anticipated difficult airway management (Mallampati class III-IV); (3) severe cardiopulmonary dysfunction precluding spontaneous ventilation (baseline SpO_2_ < 90% on room air, PaCO_2_ > 50 mmHg); (4) coagulopathy (international normalized ratio >1.5 or platelet count <50,000/μL); (5) persistent cough or excessive secretions; (6) neurological conditions affecting respiratory drive; (7) conversion to open thoracotomy; (8) incomplete outcome data.

**Non-intubated Anesthetic Protocol:**

Patient Selection Criteria for NI-UVATS:

**Inclusion criteria:**

- BMI 19–35 kg/m^2^ (recommended range; deviations permitted based on individualized multidisciplinary assessment)
- Mallampati score I–III
- Adequate pulmonary reserve (FEV_1_ > 50% predicted, PaO_2_ > 60 mmHg on room air)
- Hemodynamic stability without high-dose vasopressor support
- Cooperative patients able to maintain positioning

**Contraindications:**

- BMI <19 or >35 kg/m^2^ (relative contraindication; may be overridden by individualized risk-benefit assessment when the hemodynamic risks of general anesthesia are judged to outweigh those of non-intubated management)
- Difficult airway (Mallampati IV, previous difficult intubation)
- Severe COPD with hypercapnia (PaCO_2_ > 50 mmHg)
- Persistent cough or excessive secretions
- Neurological conditions affecting respiratory drive
- Inability to cooperate or maintain lateral position
- Anticipated procedure duration >3 h
- Expected significant bleeding risk

**Sedation Protocol:**

NI-UVATS employed conscious sedation to deep sedation based on patient tolerance:

- Initial: Midazolam 1–2 mg IV + fentanyl 25–50 μg IV
- Maintenance: Propofol infusion 25–75 μg/kg/min or dexmedetomidine 0.2–0.7 μg/kg/hr—Target: Ramsay sedation score 2–4 (responsive to verbal/tactile stimulation)
- Supplemental: Fentanyl 25 μg boluses for pain control

**Regional Anesthesia:**

- Intercostal nerve blocks: 0.5% bupivacaine 3–5 mL per space (T3–T8)
- Alternative: Paravertebral block T4–T7 with 0.375% bupivacaine 15–20 mL
- Vagal block: 2% lidocaine 2–3 mL at vagus nerve for cough suppression

**Monitoring:**

- Standard ASA monitors plus arterial line for continuous blood pressure and blood gas monitoring
- Capnography via nasal cannula or face mask
- Neuromuscular monitoring was NOT routinely used as patients maintained spontaneous ventilation
- Bispectral index (BIS) monitoring in selected cases with deeper sedation

### Conversion definition

Conversion to intubation was prospectively categorized as:—Surgical conversion: planned or unplanned intubation required due to surgical factors (bleeding, inadequate exposure, dense adhesions, or need for extended resection)—Anesthetic conversion: emergency airway rescue due to respiratory compromise (SpO_2_ < 85% unresponsive to supplemental oxygen), hemodynamic instability (sustained hypotension requiring vasopressors), failed sedation, or patient intolerance

### Data collection

Demographic, procedural and clinical data were retrospectively extracted from electronic medical records using a standardized case report form. Baseline characteristics included age, sex, body mass index, ASA classification, smoking history (pack-years), pulmonary function tests [forced expiratory volume in 1 s (FEV_1_), FEV_1_/forced vital capacity ratio], and comorbidities quantified using the Charlson Comorbidity Index (20).

Operative variables encompassed surgical approach (uniportal) procedure type, operative duration, estimated blood loss, conversion rates, and reasons for conversion. Anesthetic management details included regional anesthesia technique (intercostal nerve block, paravertebral block, or thoracic epidural), sedation protocols, intraoperative hemodynamic parameters, and arterial blood gas analyses.

### Outcome measures

The primary outcome was serious complications defined as the occurrence of any of the following within 30 days: mortality, reintubation, pneumonia, or reoperation. Secondary outcomes included individual components of the composite endpoint, pleural effusion requiring intervention, prolonged intubation (>48 h), tracheostomy, delirium, hospital length of stay, readmission within 30 days, and Surgery-related mortality at 1 year, defined as death directly attributable to the index thoracic procedure, as assessed by clinical records.

### Statistical analysis

Statistical analyses were performed using R Studio version 4.3.1 (R Foundation for Statistical Computing, Vienna, Austria) and SPSS version 28.0 (IBM Corp., Armonk, NY). Continuous variables were assessed for normality using the Shapiro–Wilk test and presented as mean ± standard deviation or median (interquartile range) as appropriate. Categorical variables were expressed as frequencies and percentages.

Between-group comparisons employed Student's *t*-test or Mann–Whitney *U* test for continuous variables and chi-square or Fisher's exact test for categorical variables. Standardized mean differences (SMD) were calculated to assess baseline balance, with SMD <0.1 indicating negligible differences between groups.

### Propensity score matching

To minimize selection bias and create comparable groups, propensity score matching was performed using the following approach:

1. **Propensity Score Calculation**: A logistic regression model was developed with treatment assignment (NI-UVATS vs. I-UVATS) as the dependent variable. Covariates included in the propensity score model were: age (continuous), sex, ASA classification (I-IV), BMI categories (<19, 19–34.9, ≥35 kg/m^2^), presence of malignancy, pulmonary disease, ischemic heart disease, diabetes mellitus, and smoking status. These variables were selected based on their potential influence on treatment selection and outcomes. Procedure type was deliberately excluded from the propensity score model as it represents a post-selection variable rather than a confounder; surgeons determine anesthetic suitability based on patient factors before selecting procedure complexity. Including mediating variables in propensity scores introduces bias and compromises causal inference. Procedural heterogeneity was instead addressed through *post-hoc* stratification by complexity (Table 6).
2. **Matching Algorithm**: We employed 1:1 nearest neighbor matching without replacement, using a caliper width of 0.1 of the standard deviation of the logit of the propensity score. This caliper of 0.1 was chosen to ensure close matches while maintaining adequate sample size. The matching was performed using the MatchIt package in R.
3. **Balance Assessment**: Covariate balance was assessed before and after matching using standardized mean differences (SMD). An SMD <0.1 was considered indicative of adequate balance between groups. Visual assessment was performed using Love plots and density plots of propensity scores.
4. **Matched Analysis**: In the matched cohort, outcomes were compared using paired tests (McNemar's test for categorical variables and Wilcoxon signed-rank test for continuous variables) to account for the matched nature of the data.

Multivariable logistic regression identified independent predictors of serious complications, incorporating variables with *p* < 0.10 in univariate analysis and clinically relevant factors based on prior literature (3). Model performance was assessed using the Hosmer-Lemeshow test and area under the receiver operating characteristic curve.

All tests were two-tailed with statistical significance defined as *p* < 0.05. Missing data patterns were evaluated, and complete case analysis was performed as missingness was <5% for all variables.

### Procedural complexity stratification

Given the heterogeneity of procedures, we performed *post-hoc* stratification by procedural complexity:

- Low complexity: Pleural/wedge biopsies, pleurodesis, simple mass resections
- medium complexity: Decortication, complex mediastinal mass resections
- High complexity: Anatomical resections (segmentectomy/lobectomy)

Stratified analyses were performed where sample sizes permitted (minimum 10 patients per group per stratum). All subgroup and stratified analyses were conducted *post-hoc* and should be considered exploratory and hypothesis-generating due to limited sample sizes within strata; findings require confirmation in larger studies.

## Results

### Patient flow and PSM

A total of 289 consecutive patients underwent UVATS procedures during the study period, with 166 (57.4%) in the I-UVATS group and 123 (42.6%) in the NI-UVATS group. After propensity score matching using a 1:1 ratio with caliper width of 0.1 standard deviation, 98 patients in each group were successfully matched and included in the final analysis. The matching process successfully balanced all baseline covariates between groups, achieving SMD <0.1 for all variables.

### Baseline characteristics before PSM

In the unmatched cohort, baseline characteristics showed generally good balance between groups with most SMD <0.1 (Table 1). Mean age was 62.5 ± 16.8 years in I-UVATS vs. 60.1 ± 15.9 years in NI-UVATS (*p* = 0.216, SMD = 0.147). Male sex distribution was comparable (48.8% I-UVATS vs. 55.3% NI-UVATS, *p* = 0.273). ASA classification revealed high-risk populations in both groups, with ASA > III present in 110 (66.3%) I-UVATS vs. 91 (74.0%) NI-UVATS patients (*p* = 0.194). Mean BMI was nearly identical between groups (26.8 ± 5.1 vs. 27.2 ± 5.3 kg/m^2^, *p* = 0.531, SMD = 0.076). Pulmonary disease was the only significant baseline difference, present in 96 (57.8%) I-UVATS vs. 99 (80.5%) NI-UVATS patients (*p* < 0.001, SMD = 0.502) representing a significant baseline imbalance. Other comorbidities were well balanced including ischemic heart disease (21.7% vs. 23.6%, *p* = 0.703), diabetes mellitus (21.1% vs. 26.8%, *p* = 0.255), and current/former smoking (42.2% vs. 49.6%, *p* = 0.210). However, the disease characteristics and the outcomes were not well balanced.

**Table 1:** Baseline patient characteristics before propensity score matching.

Characteristic	Intubated (*n* = 166)	Non-Intubated (*n* = 123)	*P*-value	SMD
Age, years				
Mea*n* ± SD	62.5 ± 16.8	60.1 ± 15.9	0.216	0.147
Male sex, *n* (%)	81 (48.8)	68 (55.3)	0.273	0.130
ASA Classification, *n* (%)				
ASA ≥ III	110 (66.26)	91 (73.98)	0.194	
Age groups, *n* (%)				
<65 years	46 (37.4)	71 (42.8)		
65–79 years	51 (41.5)	74 (44.6)		
80–89 years	22 (17.9)	18 (10.8)		
≥90 years	4 (3.3)	3 (1.8)		
BMI, kg/m^2^				
Mean ± SD	26.8 ± 5.1	27.2 ± 5.3	0.531	0.076
Median (IQR)	26.3 (23.1–29.8)	26.9 (23.5–30.4)		
BMI categories, *n* (%)				
<19 (Underweight)	10 (8.1)	11 (6.6)		
≥35 (Severely obese)	6 (4.9)	10 (6.0)		
Comorbidities, *n* (%)				
Pulmonary disease	96 (57.8)	99 (80.5)	<0.001	0.502
Ischemic heart disease	36 (21.7)	29 (23.6)	0.703	0.046
Diabetes mellitus	35 (21.1)	33 (26.8)	0.255	0.135
COPD	30 (18.1)	17 (13.8)	0.332	0.117
CRF	7 (4.2)	5 (4.1)	0.943	0.008
Current/Former smoker, *n* (%)	70 (42.2)	61 (49.6)	0.210	0.149

### Baseline characteristics after matching

In the propensity-matched cohort, baseline characteristics were well-balanced between groups (Table 1). Mean age was 61.3 ± 17.1 years in I-UVATS vs. 60.7 ± 16.3 years in NI-UVATS (*p* = 0.803, SMD = 0.036). Male sex was equally distributed (50.0% in each group). ASA classification showed similar distributions, with the majority being ASA III patients (55.1% I-UVATS vs. 57.1% NI-UVATS, *p* = 0.870). Malignancy was present in 52 (53.1%) I-UVATS vs. 55 (56.1%) NI-UVATS patients (*p* = 0.666, SMD=0.061). (Table 2). Twelve NI-UVATS patients (12.2%) were treated outside the recommended BMI range (8 with BMI <19 and 4 with BMI ≥35), based on individualized multidisciplinary assessment; no adverse outcomes were attributable to these deviations.

**Table 2:** Baseline patient characteristics after propensity score matching.

Characteristic	Intubated (*n* = 98)	Non-Intubated (*n* = 98)	*P*-value	SMD
Age, years				
Mean ± SD	61.3 ± 17.1	60.7 ± 16.3	0.803	0.036
Male sex, *n* (%)	49 (50.0)	49 (50.0)	1.000	0.000
ASA Classification, *n* (%)				
ASA III	66 (67.34)	69 (70.4)	0.870	
Age groups, *n* (%)			0.953	
<65 years	42 (42.9)	40 (40.8)		
65–79 years	40 (40.8)	44 (44.9)		
80–89 years	14 (14.3)	12 (12.2)		
≥90 years	2 (2.0)	2 (2.0)		
BMI categories, *n* (%)			1.000	
<19 (Underweight)	8 (8.2)	8 (8.2)		
≥35 (Severely obese)	4 (4.1)	4 (4.1)		
Comorbidities, *n* (%)				
Pulmonary disease	58 (59.2)	61 (62.2)	0.658	0.062
Ischemic heart disease	21 (21.4)	23 (23.5)	0.732	0.049
Diabetes mellitus	20 (20.4)	22 (22.4)	0.730	0.049
COPD	17 (17.3)	15 (15.3)	0.699	0.055
Current/Former smoker	42 (42.9)	45 (45.9)	0.667	0.061
Malignancy, *n* (%)	52 (53.1)	55 (56.1)	0.666	0.061

### Disease characteristics

In the matched cohort, malignant disease types showed balanced distribution. Among malignant cases, lung cancer was present in 25 (48.1%) I-UVATS vs. 23 (41.8%) NI-UVATS patients. Hematological malignancies occurred in 9 (17.3%) I-UVATS vs. 12 (21.8%) NI-UVATS patients. Benign diseases were similarly distributed, with pleural disease being most common in both groups (Table 3).

**Table 3:** Disease characteristics after propensity score matching.

Characteristic	Intubated (*n* = 98)	Non-Intubated (*n* = 98)	*P*-value
Malignancy, *n* (%)	52 (53.1)	55 (56.1)	0.666
Malignant disease type, *n* (%)			0.812
Lung cancer	25 (48.1)	23 (41.8)	
Metastatic disease	11 (21.2)	10 (18.2)	
Hematological malignancy	9 (17.3)	12 (21.8)	
Sarcoma	4 (7.7)	5 (9.1)	
Thymic tumor	3 (5.8)	5 (9.1)	
Benign disease type, *n* (%)			0.754
Pleural disease	23 (50.0)	19 (44.2)	
Pulmonary nodule	6 (13.0)	4 (9.3)	
Infectious	8 (17.4)	10 (23.3)	
Mediastinal mass	5 (10.9)	4 (9.3)	
Other	4 (8.7)	6 (14.0)	

### Anesthesia and surgical data after matching

In the matched cohort, anesthetic management differences persisted. I-UVATS patients had lower Mallampati scores (36.7% grade 1 vs. 24.5%, *p* = 0.019) and required more post operative oxygen supplementation (43.9% vs. 6.1%, *p* < 0.001) but less arterial line monitoring (19.4% vs. 60.2%, *p* < 0.001). Operative time remained significantly shorter in I-UVATS (median 37 vs. 52 min, *p* = 0.042) (Table 4).

**Table 4:** Anesthesia and surgical data after propensity score matching.

Characteristic	Intubated (*n* = 98)	Non-Intubated (*n* = 98)	*P*-value
Mallampati score, *n* (%)			0.019
Grade 1	36 (36.7)	24 (24.5)	
Grade 2	58 (59.2)	62 (63.3)	
Grade 3	4 (4.1)	11 (11.2)	
Grade 4	0 (0.0)	1 (1.0)	
Monitoring, *n* (%)			
Oxygen supplementation	43 (43.9)	6 (6.1)	<0.001
Arterial line	19 (19.4)	59 (60.2)	<0.001
Operation time, min			
Mean ± SD	59.8 ± 63.4	73.2 ± 65.7	0.148^a^
-Median (IQR), min	37 (20–65)	52 (30–85)	0.042^b^
Surgical procedure, *n* (%)			<0.001
Biopsy (Wedge/Pleural)	16 (16.3)	53 (54.1)	
Segmentectomy/Lobectomy	36 (36.7)	5 (5.1)	
Decortication	25 (25.5)	12 (12.2)	
Pleurodesis	9 (9.2)	10 (10.2)	
Mass resection	12 (12.2)	18 (18.4)	
Number of chest drains, *n* (%)			<0.001
Single	90 (91.8)	54 (55.1)	
Double	8 (8.2)	44 (44.9)	
Recruitment maneuvers, *n* (%)	94 (95.9)	3 (3.1)	<0.001

^a^ Student's *t*-test.

^b^ Mann–Whitney *U* test.

### Postoperative outcomes after matching

**Figure 1:**
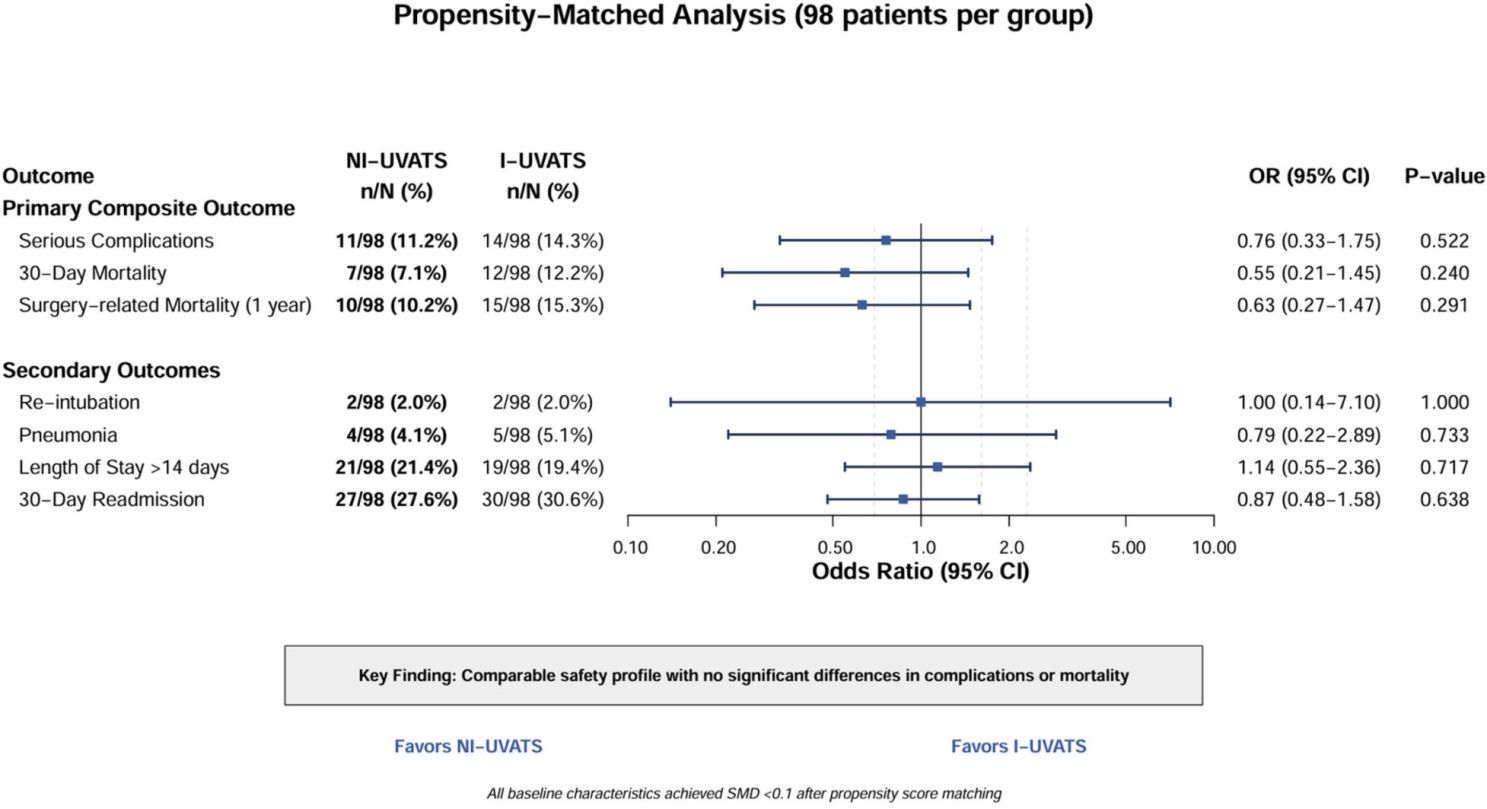
Forest plot comparing primary and secondary outcomes between non-intubated uniportal video-assisted thoracic surgery (NI-UVATS) and intubated uniportal video-assisted thoracic surgery (I-UVATS) after propensity score matching. The forest plot displays odds ratios (OR) with 95% confidence intervals (CI) for the primary composite outcome and individual secondary outcomes in 98 matched patients per group. The primary composite outcome included serious complications, 30-day mortality, and surgery-related mortality at 1 year. Secondary outcomes included re-intubation, pneumonia, length of stay >14 days, and 30-day readmission. The vertical line at OR = 1.0 represents no difference between groups. Points to the left of 1.0 favor NI-UVATS, while points to the right favor I-UVATS. *P*-values are shown for each comparison. All baseline characteristics achieved standardized mean difference (SMD) < 0.1 after propensity score matching, indicating good balance between groups. CI, confidence interval; I-UVATS, intubated uniportal video-assisted thoracic surgery; NI-UVATS, non-intubated uniportal video-assisted thoracic surgery; OR, odds ratio; SMD, standardized mean difference.

In the matched cohort, respiratory complications showed mixed patterns. While pneumonia rates remained similar (5.1% vs. 4.1%, *p* = 0.733); Pleural effusions were classified as reactive/inflammatory (78.4%), disease- related/malignant (18.6%), or postoperative hemothorax (3.1%), with similar distribution between groups (*p* = 0.412). Management included conservative observation (46.4%), extended chest tube drainage >5 days (41.2%), thoracentesis (9.3%), and re-operation for hemothorax (3.1%). Overall intervention rates were comparable between NI-UVATS (53.9%) and I-UVATS (50.0%) (*p* = 0.583). Serious complications occurred in 14 (14.3%) I-UVATS vs. 11 (11.2%) NI-UVATS patients (*p* = 0.522). Thirty-day mortality was 12 (12.2%) in I-UVATS vs. 7 (7.1%) in NI-UVATS (*p* = 0.240), and surgery-related mortality at 1 year was 10 (10.2%) vs. 15 (15.3%), respectively (*p* = 0.291) (Table 5). Figure 1 illustrates the comparative analysis of these primary and secondary outcomes, demonstrating no significant differences in odds ratios between the two approaches.

**Table 5:** Postoperative outcomes after propensity score matching.

Outcome	Intubated (*n* = 98)	Non-intubated (*n* = 98)	*P*-value
Respiratory complications, *n* (%)			
Pneumonia	5 (5.1)	4 (4.1)	0.733
Pleural effusion	34 (34.7)	63 (64.3)	<0.001
Re-intubation	2 (2.0)	2 (2.0)	1.000
Prolonged intubation	0 (0.0)	4 (4.1)	0.044
Tracheostomy	0 (0.0)	1 (1.0)	0.316
Other complications, *n* (%)			
Delirium	2 (2.0)	6 (6.1)	0.149
Postoperative pain	52 (53.1)	36 (36.7)	0.022
Serious complications, *n* (%)	14 (14.3)	11 (11.2)	0.522
Mortality, *n* (%)			
Intraoperative	2 (2.0)	2 (2.0)	1.000
30-day	12 (12.2)	7 (7.1)	0.240
Surgery-related (1 year)	10 (10.2)	15 (15.3)	0.291
Length of stay, *n* (%)			0.814
<3 days	17 (17.3)	13 (13.3)	
3–6 days	29 (29.6)	32 (32.7)	
7–14 days	33 (33.7)	32 (32.7)	
>14 days	19 (19.4)	21 (21.4)	
30-day readmission, *n* (%)	30 (30.6)	27 (27.6)	0.638

Mortality was further classified by etiology. Of 19 deaths within 30 days, 3 (15.8%) were directly procedure-related, 12 (63.2%) resulted from underlying disease progression, and 4 (21.0%) were due to unrelated medical causes. Similarly, among 25 deaths within 1 year classified as surgery-related, 5 (20.0%) were direct procedural complications, 17 (68.0%) reflected disease progression, and 3 (12.0%) were unrelated causes. The distribution of mortality causes was similar between groups (*p* = 0.385).

### Procedural complexity analysis

#### Procedural complexity distribution

Significant procedural imbalance persisted after propensity score matching. Low-complexity procedures comprised 82.7% (81/98) of NI-UVATS vs. 37.8% (37/98) of I-UVATS cases (*p* < 0.001). Conversely, high-complexity anatomical resections represented 36.7% (36/98) of I-UVATS vs. only 5.1% (5/98) of NI-UVATS procedures (*p* < 0.001). This distribution reflects contemporary practice patterns where surgeons preferentially select NI-UVATS for lower-complexity interventions. (Table 6)

**Table 6:** Outcomes stratified by procedural complexity.

Outcome	Low complexity	Medium complexity	High complexity
	I-UVATS (*n* = 37)/NI-UVATS (*n* = 81)	I-UVATS (*n* = 25)/NI-UVATS (*n* = 12)	I-UVATS (*n* = 36)/NI-UVATS (*n* = 5)
Operative time, median (IQR)	35 (20–55)/42 (25–65)	85 (55–120)/78 (45–110)	115 (85–145)/95 (70–120)^a^
Serious complications, *n* (%)	4 (10.8)/6 (7.4)	4 (16.0)/2 (16.7)	6 (16.7)/3 (60.0)^a^
30-day mortality, *n* (%)	3 (8.1)/4 (4.9)	3 (12.0)/1 (8.3)	6 (16.7)/2 (40.0)^a^
Pleural effusion, *n* (%)	21 (56.8)/24 (29.6)^b^	11 (44.0)/5 (41.7)	25 (69.4)/3 (60.0)^a^

^a^ Interpretation limited by small NI-UVATS sample size.

^b^ *p* = 0.005.

#### Outcomes stratified by procedural complexity

Among low-complexity procedures (*n* = 118), comparison of NI-UVATS (*n* = 81) vs. I-UVATS (*n* = 37) revealed no significant differences in operative time [42 [IQR: 25–65] vs. 35 [IQR: 20–55] min, *p* = 0.089], serious complications (7.4% vs. 10.8%, *p* = 0.545), or 30-day mortality (4.9% vs. 8.1%, *p* = 0.504). However, pleural effusion rates were significantly lower in I-UVATS (29.6% vs. 56.8%, *p* = 0.005).

For medium-complexity procedures, specifically decortications (*n* = 37), NI-UVATS (*n* = 12) and I-UVATS (*n* = 25) demonstrated comparable outcomes including operative time [78 [IQR: 45–110] vs. 85 [IQR: 55–120] min, *p* = 0.642], serious complications (16.7% vs. 16.0%, *p* = 0.959), and pleural effusion rates (41.7% vs. 44.0%, *p* = 0.894).

High-complexity anatomical resections were performed predominantly under I-UVATS (*n* = 36) compared to NI-UVATS (*n* = 5). Given the limited sample size in the NI-UVATS group, meaningful statistical comparison was precluded; these findings are reported descriptively only.

#### Anesthetic management

Among 98 NI-UVATS patients, sedation levels achieved included minimal sedation (Ramsay 2) in 31 patients (31.6%), moderate sedation (Ramsay 3) in 52 patients (53.1%), and deep sedation (Ramsay 4) in 15 patients (15.3%). Regional anesthesia techniques comprised intercostal nerve blocks in 76 patients (77.6%), paravertebral blocks in 18 patients (18.4%), and combined techniques in 4 patients (4.1%). Vagal nerve block was performed in 43 patients (43.9%) to minimize cough reflex during hilar manipulation.

Conversion to intubation occurred in 2 patients (2.0%), both for surgical reasons: inadequate surgical exposure (*n* = 1) and intraoperative bleeding requiring improved lung isolation (*n* = 1). Notably, no conversions occurred due to anesthetic complications such as respiratory compromise, hemodynamic instability, or failed sedation.

#### Multivariable analysis

**Figure 2:**
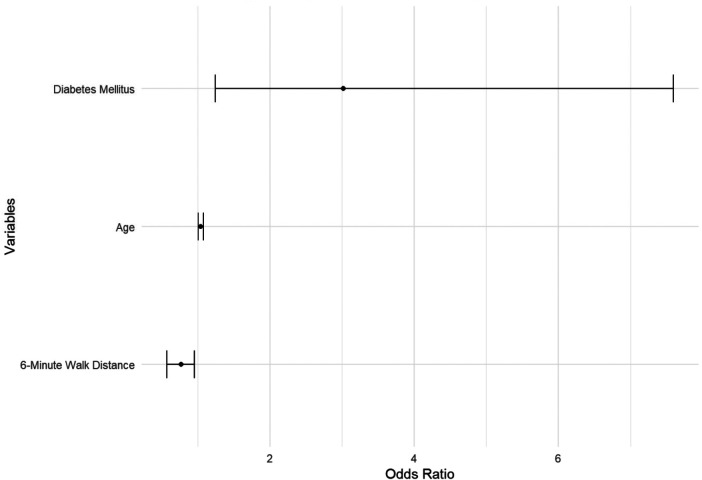
Multivariant logistic Regression—Serious Complications.

In the matched cohort, univariate analysis identified age (OR: 1.05, 95% CI: 1.02–1.08, *p* = 0.002), diabetes (OR: 2.75, 95% CI: 1.28–5.91, *p* = 0.009), and ischemic heart disease (OR: 2.12, 95% CI: 0.97–4.63, *p* = 0.059) as predictors of serious complications. In multivariable analysis, age (adjusted OR: 1.04, 95% CI: 1.01–1.08, *p* = 0.024) and diabetes (adjusted OR: 2.84, 95% CI: 1.19–6.79, *p* = 0.019) remained independent predictors of serious complications. The anesthetic approach (non-intubation) did not show a significant protective effect (adjusted OR: 0.88, 95% CI: 0.34–2.27, *p* = 0.789). Figure 2.

#### Subgroup analysis paragraph

Exploratory Subgroup Analyses The following subgroup analyses are hypothesis-generating due to limited sample sizes and should be interpreted with caution.

Subgroup analysis by age demonstrated consistent benefits of NI-UVATS across all age strata, with the most pronounced difference in pleural effusion rates. In patients younger than 65 years, pleural effusion occurred in 25.0% of NI-UVATS vs. 52.5% of I-UVATS patients (*p* = 0.012), while serious complications remained low in both groups (5.0% vs. 7.1%, *p* = 0.687). Among patients aged 65–79 years, the pleural effusion advantage persisted (31.8% vs. 65.9%, *p* = 0.002) with comparable serious complication rates (11.4% vs. 15.0%, *p* = 0.623). In the elderly population (≥80 years), although pleural effusion rates were higher overall, NI-UVATS maintained its advantage (50.0% vs. 75.0%, *p* = 0.089), despite similar rates of serious complications (25.0% vs. 28.6%, *p* = 0.826). Thirty-day mortality showed no significant differences across age groups, though a trend toward higher mortality was observed in elderly I-UVATS patients (28.6% vs. 12.5%, *p* = 0.276). Length of stay exceeding 7 days was comparable between groups across all age strata, suggesting that the recovery trajectory was not significantly influenced by the anesthetic approach regardless of patient age (Supplementary Table S1, S2; Figure 2).

Further subgroup analyses demonstrate that NI-UVATS consistently reduced operative times and postoperative pleural effusion rates across all surgery types (biopsies, decortications, and anatomical resections) and disease categories (pulmonary disease and malignancy). Despite the heterogeneity of procedures and pathologies, the benefits of NI-UVATS were maintained, with the most pronounced advantages seen in shorter operative times (31–74 min for NI-UVATS vs. 45–115 min for I-UVATS, Supplementary Table S3–S5) and reduced pleural effusion rates (29.6–39.0% for NI-UVATS vs. 57.6–73.3% for I-UVATS) across all subgroups. (Supplementary Table S6–S7)

## Discussion

This propensity-matched analysis provides real-world safety data for NI-UVATS within contemporary practice patterns rather than a head-to-head comparison across all procedure types. The marked procedural imbalance—anatomical resections comprising 36.7% of I-UVATS vs. only 5.1% of NI-UVATS cases (*p* < 0.001)—is not merely a limitation but a central finding reflecting how experienced thoracic surgeons currently apply these techniques. Surgeons predominantly select NI-UVATS for diagnostic procedures, biopsies, and pleural interventions while reserving intubated approaches for anatomical resections requiring complete lung isolation. Within this context, NI-UVATS demonstrated comparable safety for low-to-medium complexity procedures (serious complications: 7.4% vs. 10.8%, *p* = 0.545 for low- complexity; 16.7% vs. 16.0%, *p* = 0.959 for medium-complexity). Our data therefore supports NI-UVATS feasibility within current conservative selection patterns, while acknowledging that comparative effectiveness for high-complexity procedures remains undefined (see Box 1). The persistent procedural imbalance after matching should therefore be interpreted as an inherent reflection of clinical decision-making rather than a methodological shortcoming, and was addressed through complexity-stratified analysis (Table 6).

**Box 1:**
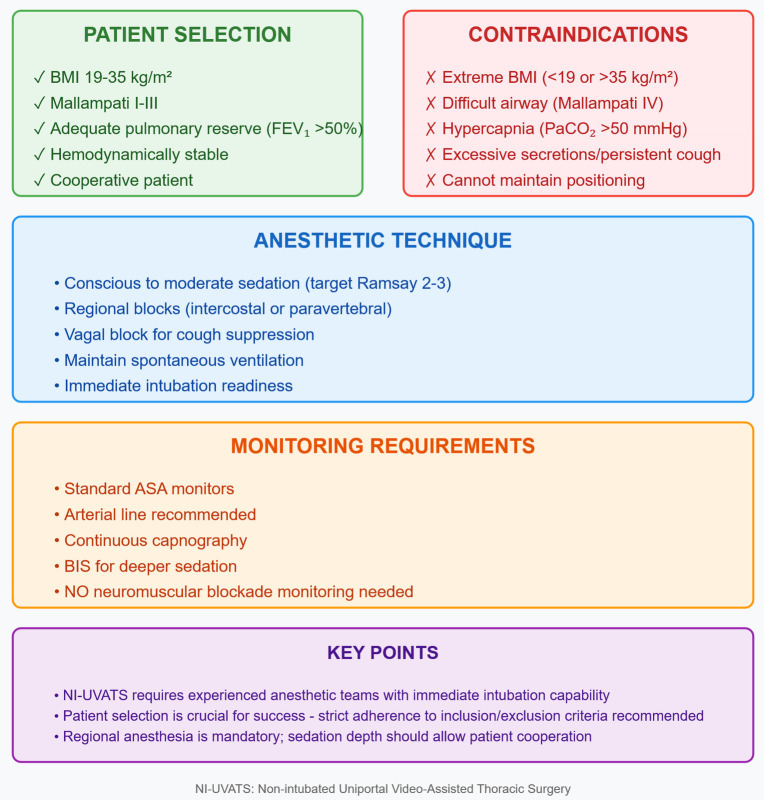
Practical considerations for NI-UVATS Implementation.

Our propensity-matched analysis of 196 patients undergoing UVATS procedures revealed comparable safety profiles between intubated and non-intubated approaches, with serious complications occurring in 14.3% vs. 11.2% of patients respectively (*p* = 0.522) which may be explained by the high rate of palliative surgery performed.

The elevated mortality rates compared to elective VATS series warrant contextualization. Our cohort included 70.4% ASA III–IV patients and 54.6% with underlying malignancy, with many procedures performed for diagnostic or palliative purposes. Importantly, mortality classification revealed that only 15.8% of 30-day deaths were directly procedure-related, while the majority (63.2%) resulted from underlying disease progression. This distribution underscores that our outcomes reflect patient selection patterns where NI-UVATS was often applied to high-risk patients who may not have tolerated conventional intubated approaches.

These findings align with recent meta-analyses demonstrating equivalent perioperative safety across diverse thoracic procedures (7, 17). The successful achievement of standardized mean differences <0.1 across all baseline characteristics after matching strengthens the validity of our comparisons and minimizes selection bias inherent in observational designs.

The comparable safety outcomes we observed support the growing body of evidence establishing NI-UVATS as a viable alternative to conventional intubated approaches. The meta-analysis by Luo et al., which focused primarily on minor VATS procedures, found no significant differences in recovery outcomes (10), while our cohort included diverse procedures with 36.7% anatomical resections in the I-UVATS group vs. 5.1% in NI-UVATS. Despite this procedural complexity differential, both approaches demonstrated similar safety profiles, reinforcing the technique's versatility.

### Technical considerations and patient selection

Our NI-UVATS protocol employed graduated sedation levels, with 84.7% of patients maintained at minimal-to-moderate sedation (Ramsay 2–3), preserving protective airway reflexes while ensuring comfort. This contrasts with some series reporting near-general anesthesia depths, supporting true “awake” thoracic surgery benefits. The absence of neuromuscular monitoring reflects our commitment to maintaining spontaneous ventilation throughout procedures. Strict patient selection criteria proved crucial for success. Our BMI limits (19–35 kg/m^2^) balanced adequate surgical exposure against respiratory compromise risks. The exclusion of Mallampati IV patients and those with baseline hypercapnia minimized conversion risk, with zero conversions for anesthetic complications. The absence of anesthetic-related conversions in our cohort validates our strict patient selection criteria and supports the safety of NI-UVATS when appropriate preoperative screening is applied. The 43.9% receiving vagal blocks experienced reduced cough reflexes, facilitating surgical precision during hilar manipulation. Regional anesthesia techniques varied by surgical approach and patient anatomy. Intercostal blocks (77.6%) provided reliable analgesia for most procedures, while paravertebral blocks offered superior coverage for extensive resections. The higher arterial line usage in NI-UVATS (60.2% vs. 19.4%) reflects our protocol for continuous respiratory monitoring during spontaneous ventilation rather than hemodynamic instability.

The physiological advantages of NI-UVATS merit consideration in interpreting our safety outcomes. Recent evidence from Patirelis et al. demonstrated that non-intubated approaches maintained diaphragmatic function better than intubated procedures (21), potentially contributing to the preserved respiratory mechanics we observed. The avoidance of positive pressure ventilation eliminates barotrauma risk and potentially reduces inflammatory mediator release as shown by Kong et al. (17, 22), which may explain the reduced oxygen supplementation requirements in NI-UVATS patients (6.1% vs. 43.9%, *p* < 0.001).

The higher pleural effusion rate observed in NI-UVATS (64.3% vs. 34.7%, *p* < 0.001) warrants discussion. As noted, the absence of positive pressure ventilation in NI-UVATS limits the ability to perform recruitment maneuvers (3.1% vs. 95.9% in I-UVATS, *p* < 0.001), resulting in incomplete alveolar re-expansion and greater residual pleural space where fluid can accumulate. Importantly, the nature of effusions was similar between groups, with the majority being reactive/inflammatory (78.4%) rather than malignant or hemorrhagic. Furthermore, despite the higher incidence, intervention rates were comparable between groups (53.9% vs. 50.0%, *p* = 0.583), and this did not translate to differences in serious complications or mortality. The higher pleural effusion rate in NI-UVATS should therefore be interpreted as a predictable physiological consequence of spontaneous ventilation—incomplete re-expansion due to absent recruitment maneuvers—rather than a clinically adverse outcome, as it did not affect the composite serious complication rate, length of stay, or 30-day readmission. Future studies should evaluate whether modified postoperative chest physiotherapy or positioning protocols can mitigate pleural effusion rates in NI-UVATS patients.

Our findings both support and extend recent randomized evidence. The 2024 non-inferiority trial by Wang et al. demonstrated comparable postoperative pulmonary complications between techniques in a highly selected cohort (11). We observed similar safety profiles regarding serious complications and mortality in an unrestricted population including 70.4% ASA III–IV patients in the NI-UVATS group, supporting the generalizability of safety outcomes to higher-risk populations previously considered unsuitable for this approach.

Operative times varied by procedural complexity. Overall, NI-UVATS required longer operative time (median 52 vs. 37 min, *p* = 0.042), which may reflect both the learning curve and need for meticulous hemostasis without muscle relaxation. For low-complexity procedures specifically, this difference was less pronounced (42 vs. 35 min, *p* = 0.089). This contrasts with the multicenter analysis by Pathonsamit et al., which reported shorter anesthesia times in NI-UVATS (16). The difference likely reflects our inclusion of more diagnostic procedures in the NI-UVATS group (54.1% vs. 16.3%, *p* < 0.001) compared to anatomical resections. Recent series have reported NI-UVATS operative times ranging from 31 to 74 min across different procedures (7, 16, 23), consistent with our findings when stratified by procedure type. As surgical teams gain experience with NI-UVATS, operative times may improve further.

The learning curve associated with NI-UVATS implementation merits particular emphasis for its educational implications. The attenuation of operative time differences from the overall cohort (median 52 vs. 37 min, *p* = 0.042) to low-complexity procedures (42 vs. 35 min, *p* = 0.089) suggests that proficiency develops more rapidly for simpler cases. Furthermore, the absence of anesthetic-related conversions in our series—with both conversions (2.0%) occurring for surgical rather than airway or hemodynamic indications—supports that adequate competency in sedation and airway management was achieved. Based on our experience, centers initiating NI-UVATS programs should consider a structured stepwise approach: beginning with low-complexity diagnostic and pleural procedures, progressing to decortications as the team gains confidence in managing spontaneous ventilation intraoperatively, and considering anatomical resections only after sufficient cumulative experience. This graduated strategy allows both surgical and anesthetic teams to develop proficiency at each complexity level before advancing, thereby minimizing patient risk during the institutional learning phase (26–29).

The phrase “expanding indications beyond traditional restrictions” in the title refers to two dimensions of our findings. First, the inclusion of patients historically excluded from NI-UVATS studies—specifically ASA III–IV patients (70.4% of the NI-UVATS cohort), elderly patients (≥65 years: 28.6%), and those with underlying malignancy (56.1%)—who were traditionally considered unsuitable candidates. Second, the broadening procedural spectrum attempted under non-intubated conditions at our center, including decortications and selected anatomical resections, beyond the diagnostic biopsies and pleurodesis to which NI-UVATS was historically confined. Our successful inclusion of elderly patients (28.6% ≥65 years) and those with significant comorbidities challenges historical limitations that reserved NI-UVATS for minor procedures in low-risk populations. The comparable serious complication rates despite this expanded application support the technique's broader feasibility. Recent evidence from Zheng et al. demonstrated that NI-UVATS lobectomies achieved similar oncological outcomes with faster recovery (22), though our data showing only 5.1% anatomical resections in NI-UVATS suggest most centers remain appropriately cautious in procedure selection while gaining experience.

Technical considerations significantly influence the successful implementation of NI-UVATS. Alternative airway management strategies, including laryngeal mask airways, have been evaluated in VATS procedures with favorable outcomes (24), though our protocol relied exclusively on spontaneous ventilation with regional anesthesia. The differential use of recruitment maneuvers (95.9% in I-UVATS vs. 3.1% in NI-UVATS, *p* < 0.001) reflects the inherent inability to perform lung recruitment during spontaneous ventilation rather than a strategic choice. However, this limitation may be physiologically balanced by the preserved diaphragmatic function during NI-UVATS, as demonstrated by Patirelis et al. (16, 21), which maintains more natural respiratory mechanics and potentially compensates for the absence of positive pressure recruitment. Our definition of intubation, which included planned conversion for specific surgical steps, differs from emergent conversion due to complications, which recent meta-analyses report at 0%–10% (7). This approach prioritizes safety while attempting to maintain spontaneous ventilation benefits during critical procedure portions, demonstrating the flexibility inherent in modern NI-UVATS protocols.

The clinical implications of our findings support NI-UVATS as a safe option for appropriately selected patients undergoing low-to-medium complexity procedures, rather than as a universal alternative to intubated approaches. NI-UVATS demonstrated reduced oxygen supplementation requirements and decreased postoperative pain (36.7% vs. 53.1% in I-UVATS, *p* = 0.022), suggesting better immediate postoperative comfort for suitable candidates. The longer median operative time in NI-UVATS [52 [IQR: 30–85] vs. 37 [IQR: 20–65] min, *p* = 0.042 by Mann–Whitney *U* test] likely reflects the learning curve and need for meticulous technique without muscle relaxation. Centers considering NI-UVATS implementation should recognize that current evidence supports its use for diagnostic procedures, pleural interventions, and selected decortications, while anatomical resections remain predominantly performed under intubation pending further prospective evaluation.

Our multivariable analysis identified age (adjusted OR: 1.04, 95% CI: 1.01–1.08, *p* = 0.024) and diabetes (adjusted OR: 2.84, 95% CI: 1.19–6.79, *p* = 0.019) as independent predictors of serious complications, while the anesthetic approach showed no significant association (adjusted OR: 0.88, 95% CI: 0.34–2.27, *p* = 0.789). This finding reinforces that patient factors rather than anesthetic technique primarily determine serious complication risk, supporting the safety of NI-UVATS when appropriate patient selection criteria are applied.

Furthermore, our exploratory subgroup analyses revealed consistent safety profiles between NI-UVATS and I-UVATS across diverse patient populations. However, these stratified findings are hypothesis-generating given the limited sample sizes within strata and require validation in adequately powered prospective studies. When stratified by age (<65 vs. ≥65 years), BMI categories (<25, 25–30, >30 kg/m^2^), underlying pathology (benign vs. malignant), and extent of surgical resection (diagnostic procedures vs. anatomical resections), we observed no significant differences in serious complication rates between the two approaches in any subgroup (all *p* > 0.05). This uniformity across subgroups strengthens the generalizability of our primary safety findings and suggests that both approaches can be safely applied across a broad spectrum of thoracic surgical patients, provided appropriate expertise and resources are available. The absence of significant interaction effects between anesthetic technique and these clinical variables further supports the non-inferiority of NI-UVATS across various clinical scenarios. The comparison of local vs. general anesthesia in specific VATS procedures, such as empyema management, has demonstrated comparable outcomes with potential benefits in select populations (25).

An important consideration is the disease heterogeneity inherent in our cohort, which included pleural procedures, diagnostic biopsies, decortications, and anatomical resections across both benign and malignant pathologies. These procedures carry distinct postoperative trajectories and survival profiles, and pooling them may obscure disease-specific effects. To address this, we performed a descriptive subanalysis restricted to patients with lung cancer (Supplementary Table S8). In this subgroup (I-UVATS *n* = 25, NI-UVATS *n* = 23), baseline characteristics and outcomes were comparable between approaches, with serious complications in 16.0% vs. 13.0% and 30-day mortality in 12.0% vs. 8.7%, respectively. However, with only 3 NI-UVATS anatomical resections among lung cancer patients, this subanalysis lacks statistical power for definitive conclusions. Our study was designed to evaluate the safety of the anesthetic approach across the full spectrum of real-world thoracic practice rather than to establish efficacy within a single disease entity. Dedicated prospective studies in homogeneous NSCLC populations undergoing curative-intent resections are needed to define the specific role of NI-UVATS in lung cancer management.

This study has several important limitations. First, despite propensity matching, significant procedural heterogeneity persisted, with anatomical resections performed almost exclusively under I-UVATS (36.7% vs. 5.1%). This imbalance precludes definitive comparative effectiveness conclusions and suggests our results primarily demonstrate NI-UVATS feasibility within current conservative selection patterns. Procedure type was intentionally excluded from the propensity model as it represents a consequence of treatment selection rather than a confounder; however, this design choice means groups were not balanced for procedural complexity, limiting head-to-head comparisons across all procedure types. Second, the small number of NI-UVATS anatomical resections (*n* = 5) prevented meaningful analysis of this technique for complex procedures. Third, our single-center design limits generalizability, as NI-UVATS requires specific anesthetic expertise. Fourth, operative time differences may reflect both procedural mix and learning curve effects, which we could not differentiate. Fifth, our sedation protocols and selection criteria may not be generalizable to all centers. The success of NI-UVATS depends heavily on experienced anesthetic teams comfortable managing spontaneous ventilation during thoracic procedures and capable of rapid conversion when necessary. Sixth, pleural effusion classification was performed retrospectively based on clinical documentation and imaging characteristics, which may have introduced some misclassification. Finally, lack of standardized selection criteria for NI-UVATS vs. I-UVATS introduces unmeasured confounding despite statistical adjustment attempts.

In conclusion, this study provides real-world evidence supporting NI-UVATS safety and feasibility for low-to-medium complexity thoracic procedures within current practice patterns. The profound procedural imbalance observed (36.7% vs. 5.1% anatomical resections) should not be viewed solely as a methodological limitation, but as valuable data reflecting contemporary surgical decision-making: experienced surgeons currently reserve NI-UVATS for lower-complexity interventions where spontaneous ventilation does not compromise surgical objectives. Our findings support NI-UVATS implementation for diagnostic procedures, pleural interventions, and selected decortications in appropriately selected patients (BMI 19–35 kg/m^2^, Mallampati I-III, adequate pulmonary reserve). The role of NI-UVATS in NSCLC management—particularly for curative-intent anatomical resections—remains undefined due to insufficient sample size in the current study and requires investigation in disease-specific prospective cohorts. Anatomical resections remain predominantly performed under intubation, and this approach appears appropriate given current evidence. Prospective, procedure-specific randomized trials are essential to define whether NI-UVATS indications can be safely expanded to complex anatomical resections.

## Supplementary Methods

Supplementary Tables S3-S5 present procedure-specific subgroup analyses from the full unmatched cohort (*n* = 289) to maximize sample size for exploratory analysis. These are complementary to Table 6, which presents complexity-stratified outcomes from the propensity-matched cohort (*n* = 196). The two sets of tables differ in source population, procedure categorization, and statistical matching—and should be interpreted independently.

## Data Availability

The raw data supporting the conclusions of this article will be made available by the authors, without undue reservation.
