# Supplementary material for: Comparative analysis of safety and outcomes of Non-intubated versus intubated uniportal video-assisted thoracic surgery using propensity score matching: a single-center experience expanding indications beyond traditional restrictions
Source: Front Surg. 2026 Apr 15;13:1798894. doi: 10.3389/fsurg.2026.1798894 (PMC13126453; doi:10.3389/fsurg.2026.1798894)
Supplement: Supplementary file 1 [file Table1.docx]

Supplementary data: **Subgroup Analysis**

**Table S1. Detailed Subgroup Analysis by Age (Exploratory Analysis – Results are Hypothesis-Generating)**

**A. Analysis by Age Groups**

| **Age Group** | **Intubated** | **Non-Intubated** | **P-value** |
| --- | --- | --- | --- |
| **<65 years (n=82)** | n=42 | n=40 |  |
| - Male sex, n (%) | 22 (52.4) | 20 (50.0) | 0.831 |
| - Serious complications, n (%) | 3 (7.1) | 2 (5.0) | 0.687 |
| - 30-day mortality, n (%) | 2 (4.8) | 1 (2.5) | 0.590 |
| - Length of stay, median (IQR) | 8 (5-12) | 7 (5-11) | 0.652 |
| **65-79 years (n=84)** | n=40 | n=44 |  |
| - Male sex, n (%) | 20 (50.0) | 22 (50.0) | 1.000 |
| - Serious complications, n (%) | 6 (15.0) | 5 (11.4) | 0.623 |
| - 30-day mortality, n (%) | 5 (12.5) | 3 (6.8) | 0.378 |
| - Length of stay, median (IQR) | 10 (6-15) | 9 (6-13) | 0.514 |
| **80-89 years (n=26)** | n=14 | n=12 |  |
| - Male sex, n (%) | 6 (42.9) | 6 (50.0) | 0.718 |
| - Serious complications, n (%) | 4 (28.6) | 3 (25.0) | 0.838 |
| - 30-day mortality, n (%) | 4 (28.6) | 2 (16.7) | 0.472 |
| - Length of stay, median (IQR) | 12 (8-18) | 11 (7-16) | 0.688 |
| **≥90 years (n=4)** | n=2 | n=2 |  |
| - Male sex, n (%) | 1 (50.0) | 1 (50.0) | 1.000 |
| - Serious complications, n (%) | 1 (50.0) | 1 (50.0) | 1.000 |
| - 30-day mortality, n (%) | 1 (50.0) | 1 (50.0) | 1.000 |
| - Length of stay, median (IQR) | 14 (10-18) | 13 (9-17) | 0.827 |

**S2. Analysis by BMI Categories** **(Exploratory Analysis – Results are Hypothesis-Generating)**

| **BMI Category** | **Intubated** | **Non-Intubated** | **P-value** |
| --- | --- | --- | --- |
| **<19 kg/m² (n=16)** | n=8 | n=8 |  |
| - Male sex, n (%) | 4 (50.0) | 4 (50.0) | 1.000 |
| - Serious complications, n (%) | 2 (25.0) | 2 (25.0) | 1.000 |
| - 30-day mortality, n (%) | 2 (25.0) | 1 (12.5) | 0.525 |
| - Length of stay, median (IQR) | 11 (7-16) | 10 (6-15) | 0.721 |
| **19-34.9 kg/m² (n=172)** | n=86 | n=86 |  |
| - Male sex, n (%) | 43 (50.0) | 43 (50.0) | 1.000 |
| - Serious complications, n (%) | 11 (12.8) | 8 (9.3) | 0.465 |
| - 30-day mortality, n (%) | 9 (10.5) | 5 (5.8) | 0.270 |
| - Length of stay, median (IQR) | 10 (6-14) | 9 (5-13) | 0.482 |
| **≥35 kg/m² (n=8)** | n=4 | n=4 |  |
| - Male sex, n (%) | 2 (50.0) | 2 (50.0) | 1.000 |
| - Serious complications, n (%) | 1 (25.0) | 1 (25.0) | 1.000 |
| - 30-day mortality, n (%) | 1 (25.0) | 1 (25.0) | 1.000 |
| - Length of stay, median (IQR) | 12 (8-17) | 11 (7-16) | 0.785 |

**Subgroup Analysis by Type of Surgery** **(Exploratory Analysis – Results are Hypothesis-Generating)** **Full cohort before propensity score matching (n=289)**

**S3. Biopsy Procedures (Full cohort before propensity score matching, n=289)**

| **Variable** | **Intubated (n=27)** | **Non-intubated (n=66)** | **p-value** |
| --- | --- | --- | --- |
| Age, mean (SD) | 63.1 (17.8) | 66.8 (19.2) | 0.389 |
| Surgery duration (min) | 31 (12–75) | 45 (22–135) | 0.021 |
| Blood loss >200ml | 3 (11.1) | 2 (3.0) | 0.282 |
| Outcomes |  |  |  |
| Serious complications | 2 (7.4) | 10 (15.2) | 0.492 |
| 30-day mortality | 2 (7.4) | 9 (13.6) | 0.618 |
| Pleural effusion | 8 (29.6) | 38 (57.6) | 0.024 |
| Length of stay >7 days | 10 (37.0) | 39 (59.1) | 0.081 |

**S4. Decortication Procedures (Full cohort before propensity score matching, n=289) — Exploratory, Hypothesis-Generating**

| **Variable** | **Intubated (n=41)** | **Non-intubated (n=15)** | **p-value** |
| --- | --- | --- | --- |
| Age, mean (SD) | 64.7 (15.2) | 69.3 (16.4) | 0.329 |
| Surgery duration (min) | 53 (20–132) | 85 (45–154) | 0.038 |
| Blood loss >200ml | 6 (14.6) | 2 (13.3) | 1.000 |
| Outcomes |  |  |  |
| Serious complications | 5 (12.2) | 4 (26.7) | 0.362 |
| 30-day mortality | 3 (7.3) | 3 (20.0) | 0.351 |
| Length of stay >7 days | 24 (58.5) | 12 (80.0) | 0.235 |

**S5. Anatomical Resections — Wedge/Segmentectomy/Lobectomy (Full cohort before propensity score matching, n=289) — Exploratory, Hypothesis-Generating**

| **Variable** | **Intubated (n=85)** | **Non-intubated (n=32)** | **p-value** |
| --- | --- | --- | --- |
| Age, mean (SD) | 66.4 (15.9) | 70.1 (15.6) | 0.262 |
| Surgery duration (min) | 74 (30–202) | 115 (58–220) | 0.003 |
| Blood loss >200ml | 12 (14.1) | 3 (9.4) | 0.714 |
| Outcomes |  |  |  |
| Serious complications | 11 (12.9) | 8 (25.0) | 0.195 |
| 30-day mortality | 7 (8.2) | 6 (18.8) | 0.195 |
| Pleural effusion | 28 (32.9) | 22 (68.8) | 0.001 |
| Length of stay >7 days | 46 (54.1) | 24 (75.0) | 0.065 |

**Note: Procedure categorization in Tables S3–S5 differs from the complexity stratification in Table 6. Tables S3–S5 classify procedures by surgical type (biopsy, decortication, anatomical resection including wedge resections), whereas Table 6 stratifies by procedural complexity (low, medium, high). Direct cross-referencing of sample sizes between these tables is therefore not appropriate.**

**Subgroup Analysis by Disease Type**

**S.6 Patients with Pulmonary Disease** **(Exploratory Analysis – Results are Hypothesis-Generating)**

| **Variable** | **Intubated (n=99)** | **Non-intubated (n=96)** | **p-value** |
| --- | --- | --- | --- |
| Age, mean (SD) | 67.85 (17.72) | 65.45 (16.12) | 0.321 |
| Male sex, n (%) | 49 (49.5) | 43 (44.8) | 0.615 |
| **Outcomes** |  |  |  |
| Serious complications | 19 (19.2) | 10 (10.4) | 0.131 |
| 30-day mortality | 15 (15.2) | 8 (8.3) | 0.209 |
| Length of stay >7 days | 68 (68.7) | 50 (52.1) | 0.025 |

**S 7. Patients with Malignancy** **(Exploratory Analysis – Results are Hypothesis-Generating)**

| **Variable** | **Intubated (n=84)** | **Non-intubated (n=84)** | **p-value** |
| --- | --- | --- | --- |
| Age, mean (SD) | 69.21 (17.38) | 66.82 (15.71) | 0.351 |
| **Tumor Location** |  |  | 0.084 |
| - Lung | 29 (34.5) | 40 (47.6) |  |
| - Other sites | 55 (65.5) | 44 (52.4) |  |
| **Outcomes** |  |  |  |
| Serious complications | 17 (20.2) | 11 (13.1) | 0.305 |
| 30-day mortality | 16 (19.0) | 9 (10.7) | 0.191 |
| Surgery-related mortality | 19 (22.6) | 11 (13.1) | 0.159 |
| Pleural effusion | 54 (64.3) | 30 (35.7) | <0.001 |

**Supplementary Table S8. Descriptive Subanalysis of Patients with Lung Cancer After Propensity Score Matching (Exploratory — Hypothesis-Generating)**

| **Variable** | **I-UVATS (n=25)** | **NI-UVATS (n=23)** |
| --- | --- | --- |
| **Baseline** |  |  |
| Age, mean ± SD | 66.2 ± 14.8 | 64.5 ± 15.1 |
| Male sex, n (%) | 14 (56.0) | 13 (56.5) |
| ASA ≥III, n (%) | 18 (72.0) | 17 (73.9) |
| **Procedure type, n (%)** |  |  |
| - Biopsy (wedge/pleural) | 8 (32.0) | 14 (60.9) |
| - Segmentectomy/Lobectomy | 15 (60.0) | 3 (13.0) |
| - Other | 2 (8.0) | 6 (26.1) |
| **Outcomes** |  |  |
| Serious complications, n (%) | 4 (16.0) | 3 (13.0) |
| 30-day mortality, n (%) | 3 (12.0) | 2 (8.7) |
| Surgery-related mortality (1 year), n (%) | 3 (12.0) | 3 (13.0) |
| Pleural effusion, n (%) | 10 (40.0) | 13 (56.5) |
| Length of stay >7 days, n (%) | 14 (56.0) | 12 (52.2) |
